# Dosing, Toxicity and Drug Concentrations for Ganciclovir/Valganciclovir in Preterm and Low Birthweight Infants Treated for Cytomegalovirus

**DOI:** 10.1097/INF.0000000000004605

**Published:** 2024-11-13

**Authors:** Asrar Abu Bakar, Helen Payne, Neil Tickner, Muhd Alwi Muhd Helmi, Tom G. Jacobs, Hermione Lyall

**Affiliations:** From the * Department of Paediatrics, International Islamic University Malaysia, Kuantan, Malaysia; † Department of Paediatric Infectious Diseases, Imperial College Healthcare NHS Trust, London, United Kingdom; ‡ Department of Pharmacy, Research Institute for Medical Innovation, Radboud University Medical Center, Nijmegen; § Department of Pharmacy, Tergooi Medical Center, Hilversum, The Netherlands.

**Keywords:** cytomegalovirus, congenital, postnatal, ganciclovir, toxicity

## Abstract

**Background:**

There is a lack of data regarding suitable dosage when administering intravenous ganciclovir (GCV) or oral valganciclovir (valGCV) to preterm and low birthweight infants with cytomegalovirus (CMV) disease.

**Methods:**

Data were collected for infants born before 32 weeks gestation and/or weighing less than 1.8 kg treated for CMV disease with GCV or valGCV between 2016 and 2023.

**Results:**

Twenty-four infants (58% males and 48% Asian ethnicity) with a median gestation of 31 weeks [interquartile range (IQR): 26.6–36.1], median weight of 950 g (IQR: 470–1692) and median age of 45 days (IQR: 6–84) at initiation of treatment were included. Seventeen infants were treated for symptomatic postnatal CMV and 7 for symptomatic congenital CMV. Most infants receiving GCV had 6 mg/kg twice daily dosing and most receiving valGCV had 16 mg/kg twice daily dosing. Fourteen infants had drug concentrations measured with combined geometric mean minimum blood plasma concentration (C_min_) of 2.44 mg/L and maximum blood plasma concentration of 7.98 mg/L for doses of 6 mg/kg GCV and 16 mg/kg valGCV, which is higher compared with term infants. The estimated area under the curve at 12 hours (AUC_0–12h_) was 54.34 mg × h/L, which doubled the value for term infants in a previous study. Notably, AUC_0–12h_ had an inverse relationship with gestational age and weight. Infants with lower gestation and higher C_min_ showed a higher tendency for more than 1 adverse effect.

**Conclusions:**

GCV and valGCV use among preterm and very low birthweight infants with CMV disease resulted in a higher incidence of adverse events, increased AUC_0–12h_ and elevated C_min_ compared with term infants. Further pharmacokinetic studies are necessary to determine the ideal dosage in this population.

Cytomegalovirus (CMV), a member of human herpesviruses, causes significant disease in infants and immunocompromised individuals. Globally, congenital cytomegalovirus (cCMV) is the most prevalent congenital infection with an estimated prevalence of 6.7 per 1000 live births and higher in lower income populations.^1,2^ Approximately 12.7% of newborns infected with CMV manifest symptoms at birth leading to permanent sequelae including hearing loss (12%) and neurocognitive impairments (5%–15%).^3–6^ Preterm infants with cCMV are at higher risk of hearing difficulties, abnormal neuroimaging, poor motor development along with growth deficiencies.^7–10^ Postnatal cytomegalovirus infection (pCMV) commonly occurs via breast milk from CMV-seropositive mothers and may lead to end-organ diseases such as marrow suppression, hepatitis, pneumonitis, colitis and sepsis-like syndrome including in preterm and very low birthweight (VLBW) infants.^11–14^ Such susceptibility warrants careful consideration for antiviral treatment yet data in preterm and VLBW remains lacking.

Ganciclovir (GCV) is an antiviral nucleoside analog used in treating CMV disease. It inhibits viral DNA through the intercalation of the molecule into viral DNA, thus blocking DNA chain elongation.^15^ Intravenous GCV has in-vitro activity against human CMV and is efficacious against infections in immunocompromised patients.^16^ The oral drug form of valganciclovir (valGCV), an L-valyl ester prodrug, undergoes rapid conversion to GCV by intestinal and hepatic esterases and is licensed by the Food and Drug Administration for use in high-risk transplant patients.^17^ However, both GCV and valGCV have been widely adopted for the treatment of symptomatic CMV disease in term infants owing to their modest benefits on hearing and neurodevelopmental outcomes.^18–20^

However, GCV induces hematologic toxicity as it affects the growth rate constant for both granulocyte and erythroid progenitors.^21^ Significant neutropenia was observed in 17%–40% of treated infants.^18,22^ Other adverse effects include transient thrombocytopenia and transaminitis although their prevalence in preterm infants is less clear. Onset of toxicity can occur within days of starting treatment and is most problematic within the first 6 weeks.^19^ Risk of adverse effects is lower for valGCV than GCV.^23,24^ Animal studies have revealed potential long-term effects of mutagenicity and impaired fertility, but there have been no reports in exposed infants to date.^25^

Determining the optimal GCV and valGCV dosage for use in preterm and VLBW infants remains unresolved due to a lack of published data on the appropriate dosing regimen, safety profile and the desired drug concentration. Pragmatic dosing of symptomatic preterm infants has relied on pharmacokinetic (PK) data obtained from infants treated beyond 32 weeks gestation and those weighing more than 1.8 kg.^19^ To date, only case reports have been published regarding the treatment of CMV disease in this vulnerable group.^26,27^ Achieving appropriate dosing for preterm infants is challenging, necessitating individualized therapeutic drug monitoring (TDM) as highlighted by a report involving premature twins.^27^ This study aims to understand the safety, PK, and tolerability of GCV and valGCV in preterm and VLBW infants to inform future dosing strategies in this vulnerable population.

## MATERIALS AND METHODS

### Study Population and Design

This retrospective study was conducted at a single tertiary hospital with approximately 9000 births annually and around 290 preterm deliveries involving infants less than 32 weeks gestation.^28^ Data on infants born before 32 weeks gestation or birthweight below 1800 g, and treated for CMV disease with GCV and/or valGCV, between 2016 and 2024 were extracted from the dispensary registry. This study was registered as clinical audit within the institution.

### Data Collection

The study involved a review of electronic clinical records. Data extracted included demographics, treatment indication, duration, dosage, GCV concentrations and documented adverse effects. Ethnicity was categorized according to White Caucasian, Asian, Afro-Caribbean or other. Gestational age and weight upon initiation of treatment were labeled as day 1 (D1). Indication for treatment was categorized as symptomatic pCMV or symptomatic cCMV disease.

Dosing was categorized to either 6 mg/kg twice daily (or other) for GCV and 16 mg/kg twice daily (or other) for valGCV. Adverse effect profile including neutropenia, thrombocytopenia, anemia and transaminitis was determined from clinical records and blood parameters before and after treatment initiation, extending up to 28 days of treatment to account for repeat dose toxicity.^21^ Infants exhibiting alternative causes for these occurrences (ie, infection) as per clinical documentation were excluded.

Blood samples for measurement of GCV were collected per local TDM protocol. The samples were typically obtained before the next dose, known as minimum blood plasma concentrations (C_min_). Samples were drawn at a steady state, at least 3 days after GCV or valGCV initiation. Postdose samples were collected either 1 hour after the beginning of GCV infusion or 2 hours after valGCV enteral administration as per TDM recommendations.^29^ We considered these samples to be a surrogate parameter for the maximum blood plasma concentration (C_max_).

### PK Calculation and Statistical Analysis

The population geometric mean (GM, %coefficient of variation) C_min_, C_max_ and estimated area under the curve at 12 hours (AUC_0–12h_) were determined. Estimated AUC_0–12h_ was calculated using the linear-up, log-down trapezoidal method. The predose concentrations were equaled to 12-hour concentrations to facilitate the calculation of AUC_0–12h_. All data were reported descriptively on an individual level as well as aggregated per GCV and valGCV dose. Regarding the target PK, we accepted an AUC_0–12h_ range of 20–55 mg × h/L with GM of 27.4 mg × h/L as highlighted in the only available study closest to our population.^23^ Statistical analyses were performed using IBM SPSS version 29 (IBM Corp, Armonk, NY). Due to nonnormal distribution of PK data, results were log-transformed before analysis. Relationships between gestational age at D1, weight at D1 and adverse events with C_min_ and AUC_0–12h_ were analyzed using Spearman’s rank correlation. Statistical significance was defined by *P*-value <0.05.

## RESULTS

Between January 2016 and February 2024, information on 24 infants was retrieved from the GCV and valGCV dispensing database. The median gestational age at birth was 24.2 weeks [interquartile range (IQR): 22.9–30.9] and the median birthweight was 591.5 g (IQR: 433–1300). There were 14 males and 10 females. Eleven infants (48%) were of Asian ethnicity, 6 White Caucasian, and 5 Afro-Caribbean with 2 defined as “other” (Table 1).

**TABLE 1.** Summary of study cohort: preterm infants ≤32 weeks of gestational age and/or weighing ≤1.8 kg

	cCMV (n/%)	pCMV (n/%)
Gender		
Male	3/24 (13)	11/24 (46)
Female	4/24 (17)	6/24 (25)
Ethnicity		
White Caucasian	1/24 (4)	5/24 (21)
Asian	5/24 (21)	6/24 (25)
Afro-Caribbean	1/24 (4)	4/24 (17)
Other	-	2/24 (8)
Median gestation at birth (weeks)	26.3 (IQR: 23.9–30.9)	24 (IQR: 22.9–27.7)
Median birthweight (g)	972 (IQR: 433–1300)	540 (IQR: 464–848)
Indication for treatment		
Symptomatic cCMV	7/24 (29)	-
Symptomatic pCMV (reason below*)	-	17/24 (71)
Thrombocytopenia	-	12/17 (71)
Pneumonitis	-	7/17 (41)
Colitis	-	4/17 (24)
Hepatitis	-	3/17 (18)
Sepsis-like syndrome	-	3/17 (18)
At onset of treatment (D1)		
Median gestation (weeks)	28.9 (IQR: 26.6–31.6)	31.4 (IQR: 27.3–36.1)
Median postnatal age (days)	10 (IQR: 2–39)	53 (IQR: 29–84)
Median weight (g)	922.5 (IQR: 470–1420)	936 (IQR: 670–1692)
Dose of treatment (D1)†		
GCV 6 mg/kg BD	4/24 (17)	6/24 (25)
GCV other BD	-	1/24 (4)
valGCV 16 mg/kg BD	3/24 (13)	7/24 (29)
valGCV other BD	-	3/24 (13)
Duration of treatment		
2 weeks	-	9/17 (53)
4 weeks	-	6/17 (35)
8 weeks	-	2/17 (12)
6 months	6/7 (86)	-
Other	1/7 (14)	-
Adverse effects‡		
Neutropenia	11/24 (46)	-
Anemia	10/24 (42)	-
Thrombocytopenia	4/24 (17)	-
Transaminitis	4/24 (17)	-
None reported	6/24 (25)	-
>2 adverse effects	10/24 (42)	-

* Nine infants (53%) exhibited 2 or more clinical indications for pCMV treatment.

† One infant received GCV 5 mg/kg dose, 3 infants received valGCV 8, 14 and 15 mg/kg.

‡ Neutropenia was defined as an absolute neutrophil count <1.0 × 10^9^/L, anemia as hemoglobin <9 g/dL, thrombocytopenia as platelet count <150 × 10^9^/L, and transaminitis as alanine aminotransferase levels exceeding twice the upper limit of normal, occurring within 28 days of treatment.

BD indicates twice daily

Seventeen infants received treatment for symptomatic pCMV. Indications for treatment included symptomatic thrombocytopenia (71%), pneumonitis or respiratory deterioration (41%), gastrointestinal problems or colitis (24%), hepatitis (18%) and sepsis-like symptoms (18%). Nine infants (53%) exhibited 2 or more clinical indications. Treatment duration for pCMV varied as follows: 9 infants received 2 weeks of treatment, 6 infants received 4 weeks of treatment and 2 infants received 8 weeks of treatment. The remaining 7 infants received treatment for symptomatic cCMV. Of these, 6 underwent a 6-month therapy regimen, whereas 1 infant died due to preterm complications and did not complete therapy (Table 1).

Nearly all infants (91%) showed an improvement in CMV viraemia after completing treatment, with data lacking in 2 infants. On average, the time to achieve viral suppression, defined as a reduction of viral load to less than 2.5 log was 19 days (IQR: 6–35 days). A rapid virological response, defined as viral load of less than 2.5 log within 14 days of initiating treatment was observed in 6 infants (25%).

At the onset of treatment (D1), the median gestational age for all infants was 31 weeks (IQR: 26.6–36.1) with a median postnatal age of 45 days (IQR: 2–84), had a median weight of 950 g (IQR: 470–1692). When stratified according to pCMV and cCMV status, the median gestational age at D1 was 31.4 weeks (IQR: 27.3–36.1) for pCMV and 28.9 weeks (IQR: 26.6–31.6) for cCMV infants. Median weights at D1 were 936 g (IQR: 670–1692) for pCMV and 922.5 g (IQR: 470–1420) for cCMV, respectively.

Twelve infants (50%) received GCV, with 11 receiving a dose of 6 mg/kg twice daily, and 1 receiving a dose of 5 mg/kg twice daily (Table 1). Twenty-one infants (88%) received valGCV, with 19 receiving 16 mg/kg twice daily, 1 receiving 14 mg/kg twice daily and another 15 mg/kg twice daily. Due to high GCV C_min_ concentrations in preceding dosages, 2 infants (P2 and P6) had dose reductions, 1 receiving 14 mg/kg twice daily and another 8 mg/kg twice daily. Nine infants (38%) sequentially received both GCV and valGCV during treatment.

Six infants (25%) experienced no documented adverse effects within 28 days of starting treatment (Table 1). Upon treatment, 11 infants (46%) developed neutropenia, 10 (42%) anemia and 4 (17%) thrombocytopenia. Transaminitis developed in 4 infants (17%). More than half (56%) of the 18 infants who developed adverse effects, had 2 or more adverse effects during the first 28 days of treatment. Two infants had treatment temporarily halted due to severe neutropenia (absolute neutrophil count < 0.5 × 10^9^/L). Neither received granulocyte colony-stimulating factor (G-CSF) and treatment was resumed successfully after improvement in neutrophil counts. One infant experienced neutropenia and received G-CSF, and subsequently completed treatment as planned.

**FIGURE 1.**
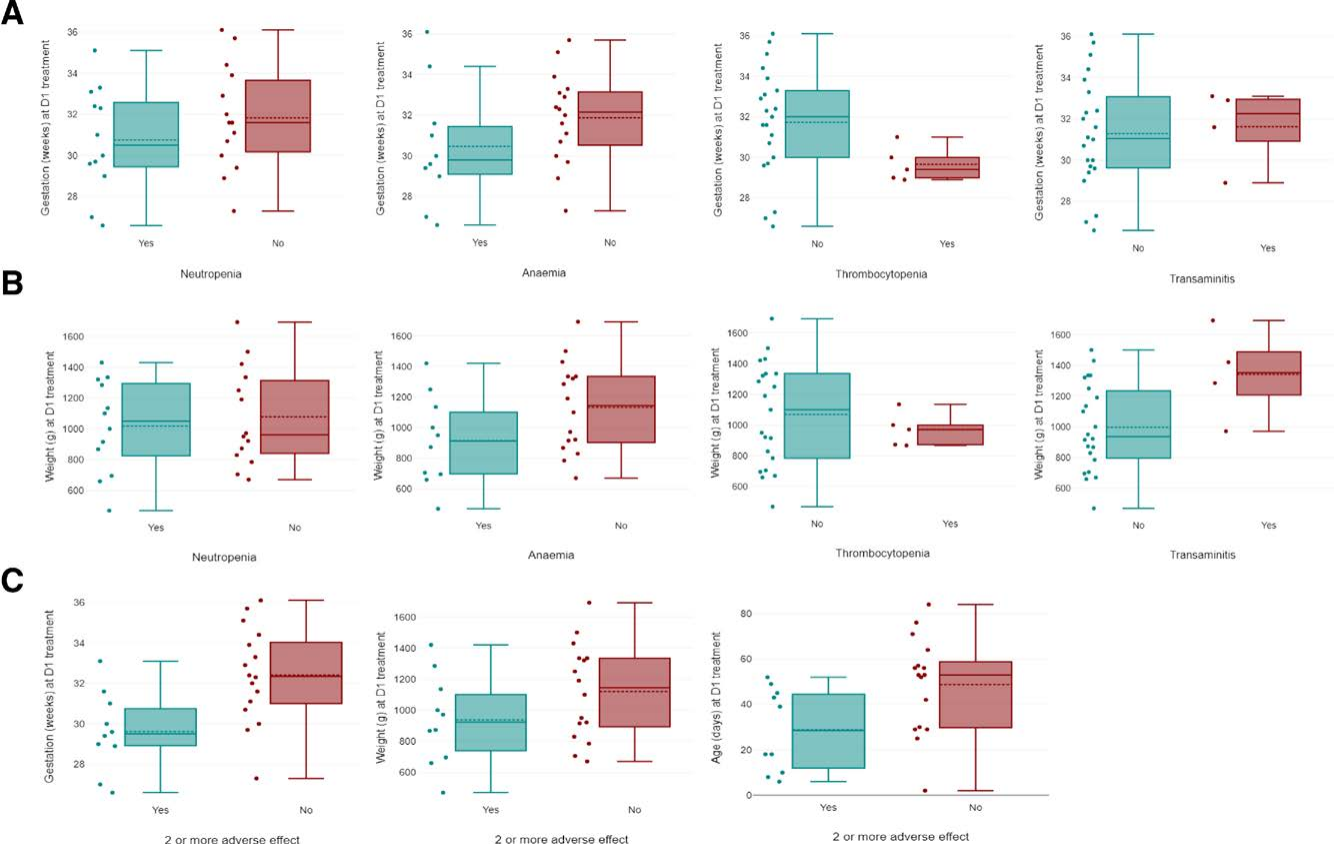
Occurrence of individual and multiple adverse events in relation to age and weight at D1. The red and green dots represent the individual gestation at D1. The solid lines in the boxplots represent the median values and the dotted lines represent the means. The boxplot shows the distribution in quartiles ranging from lowest to the highest gestation (weeks) at D1 (section A), weight (g) at D1 (section B) and its relation with the development of neutropenia, anemia, thrombocytopenia and transaminitis separately. In section C, the boxplot shows the distribution between the lowest and highest value for gestation (weeks), weight (g) and chronological age (days) at D1 in relation to multiple adverse effects. No significant correlation was seen between gestational age and the development of these adverse effects in section A. No significant correlation was seen between weight and development of neutropenia, anemia and thrombocytopenia except transaminitis (r = 0.41, *P =* 0.03) in section B. Both gestation (r = −0.45, *P =* 0.01) and age (r = −0.42, *P =* 0.02) had a negative correlation with the development of 2 or more adverse effects in section C.

There were no significant correlations among prevalence of neutropenia, anemia or transaminitis with gestational age or weight at D1 (Fig. 1). However, heavier infants had more transaminitis [*P* = 0.036, 95% confidence interval (CI): −666.2 to −23.9]. Infants with 2 or more adverse effects tended to have lower gestational (*P* = 0.011, 95% CI: −4.64 to −0.94) and chronologic age at D1 (*P* = 0.023, 95% CI: −36.8 to −2.98) but no significant differences in weight (Fig. 1).

For GCV concentrations, measurements were obtained for 14 infants (58%) (Table 2). The combined C_min_ (GM: 2.44 mg/L) was higher compared with term infants (GM: 0.60 mg/L) receiving GCV 6 mg/kg, whereas the C_max_ was only slightly higher (GM: 7.98 mg/L) compared with term infants (GM: 6.02 mg/L) in the previous study.^27^ One infant had their dose reduced to 8 mg/kg valGCV twice daily following a C_min_ of 4.9 mg/L on 16 mg/kg valGCV twice daily. The subsequent C_min_ following the dosing change was 2.6 mg/L. The infant was later switched to 16 mg/kg valGCV with a C_min_ of 1.1 mg/L, highlighting the evolving renal clearance with age in these infants.

**Table 2.** Individual dosages, drug concentrations and toxicity

Patient	Dose of GCV (mg/kg BD)	Dose of valGCV (mg/kg BD)	GCV	GCV	valGCV	valGCV	AUC_0–12h_ (mg × h/L)	Adverse effect(s)
			C_min_ (mg/L)	C_max_ (mg/L)	C_min_ (mg/L)	C_max_ (mg/L)		
P1	6	16	3.3	7.3			59.07	Neutropenia, Anemia
P2*	6	16	7	11.8			107.01	Neutropenia, Anemia
P2		14			2.8			
P3	6	16	3.5	12.5			84.02	Thrombocytopenia, Hepatitis
P4		16						Neutropenia, Anemia, Thrombocytopenia
P5		16						Anemia, Thrombocytopenia
P6†		16			4.9	10.2	82.49	Neutropenia (given G-CSF)
P6		8			2.6			
P6		16			1.1	8.6	45.07	
P7	6	16	3.4	8.5			65.48	Anemia, Hepatitis
P8	6							None reported
P9		16						Neutropenia, Anemia
P10	6		2.2					Anemia
P11	6	16						Neutropenia, Thrombocytopenia
P12		16			1.7	10.6	59.23	None reported
P13	6	16	1.6	4.6			33.55	Neutropenia, Anemia, Thrombocytopenia
P14		16				7.7		None reported
P15		16						None reported
P16	5							None reported
P17		14			3.5	9.9	71.45	Neutropenia
P18	6	16						Neutropenia
P19	6	16	0.2	2.8			12.24	Hepatitis
P20		15						Neutropenia, Hepatitis
P21		16			5.9			Anemia
P22		16						Neutropenia
P23		16						None reported
P24	6	16						Anemia

* P2 had a dose reduction to 14 mg/kg following a high C_min_ reading on 16 mg/kg dose, and a subsequent lower C_min_ on the lower dose.

† P6 highlights the evolving renal clearance depicted by variations in C_min_ and C_max_ values over time as highlighted in the main text.

BD indicates twice daily.

**Figure 2.**
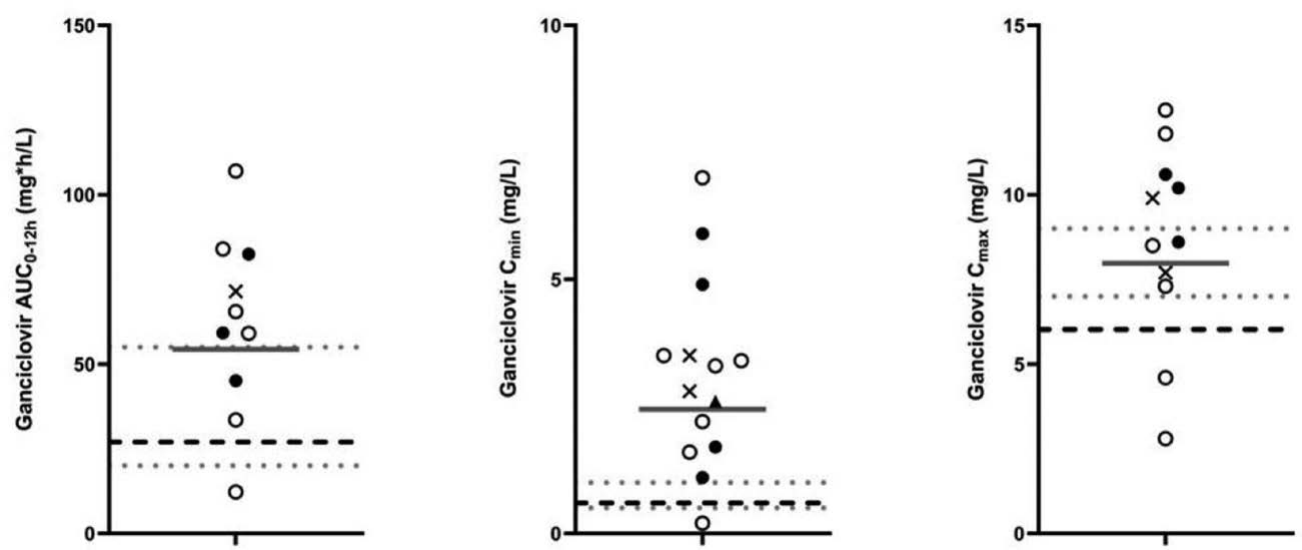
Individual ganciclovir concentrations—AUC_0–12h_, C_min_, and C_max_. Left panel: ganciclovir AUC_0–12h_; middle panel: ganciclovir C_min_; right panel: ganciclovir C_max_. Gray solid lines represent the geometric means, gray dotted lines represent the neonatal AUC_0–12h_ target range as reported by Kimberlin and colleagues and the adult target C_max_ and C_min_ ranges as reported by Luck and colleagues. Black dashed lines represent the median AUC_0–12h_, C_max_, and C_min_ for neonates receiving 6 mg/kg ganciclovir as reported by Trang and colleagues and Kimberlin and colleagues. Open dots represent infants receiving GCV 6 mg/kg; black dots represent infants receiving valGCV 16 mg/kg; crosses represent infants receiving valGCV 14 mg/kg; black triangle represents an infant receiving valGCV 8 mg/kg.

Of the 14 infants, 10 infants had both pre- and postdose drug levels, allowing for estimates of the AUC_0–12h_ (Table 2). The AUC_0–12h_ values for GCV at 6 mg/kg and valGCV at 16 mg/kg yielded a GM of 49.25 and 60.38 mg × h/L, respectively. The overall combined GM for all infants with the above dosages was 54.34 mg × h/L (CV = 68%), which is almost 2-fold higher compared with the GM AUC_0–12h_ reference value of 27.4 mg × h/L in term infants receiving a similar dose of valGCV.^22^ Specifically, 7 (70%) of the infants with PK data had values exceeding the upper range AUC_0–12h_ limit of 55 mg × h/L (IQR: 12.23–107.01 mg × h/L) (Fig. 2).

**Figure 3.**
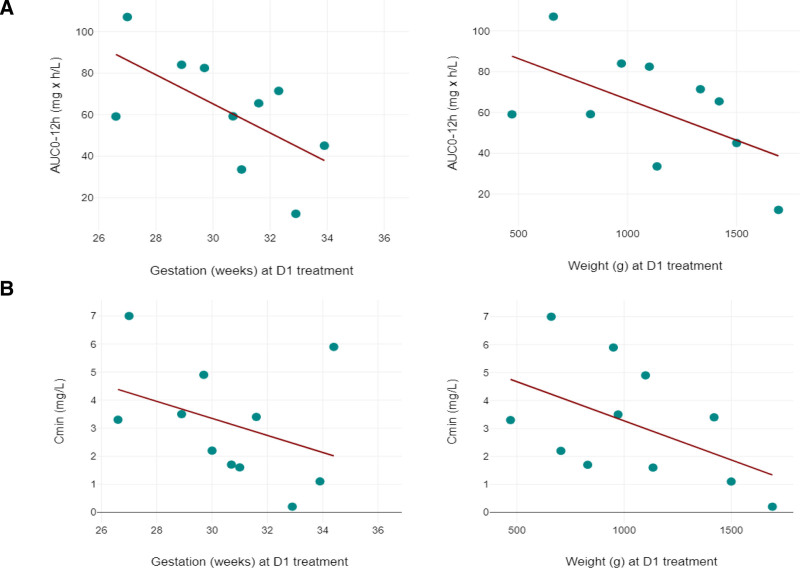
AUC_0–12h_ and C_min_ for gestation and weight at D1. The dots represent individual AUC_0–12h_ and C_min_ levels in relation to gestation and weight. Section A represents the AUC_0–12h_ in relation to gestational age at initiation of treatment and to weight. AUC_0–12h_ has a negative, moderate correlation with gestation at D1 (r = −0.55, *P* = 0.049) and nonsignificant correlation with weight at D1 (r = −0.5, *P* = 0.069). Section B represents the C_min_ in relation to gestational age at initiation of treatment and to weight. C_min_ has a negative, weak nonsignificant correlation with gestation at D1 (r = −0.39, *P* = 0.235) and weight at D1 (r = −0.53, *P* = 0.096).

No significant relationship was observed between gender, ethnicity and AUC_0–12h_. Gestational age at D1 displayed a substantial negative correlation with AUC_0–12h_ (Spearman, r = −0.55, *P* = 0.049), whereas weight at D1 also showed a negative but none significant correlation (Spearman, r= −0.5, *P* = 0.069) (Fig. 3). A similar but less significant correlation is also seen with C_min_ (Fig. 3). The cCMV group exhibited higher values for AUC_0–12h_ (GM 71.1 mg × h/L, CV = 29.6%) compared with the pCMV group (GM 36.3 mg × h/L, CV = 60%), owing to the cCMV group having lower gestational age and weights at D1 (median gestation: 29.3 vs. 31.7 weeks and median weight: 1036 vs. 1235 g).

Six (86%) of 7 infants with higher than proposed upper limit of AUC_0–12h_ had adverse effects, and in more than half (57%), there were 2 or more. Two of three infants with AUC_0–12h_ within the 20–55 mg × h/L range had adverse effects but only one had 2 or more adverse effects. Infants with neutropenia and anemia had higher C_min_ values (GM: 4.2 vs. 2.6 mg/L, and GM: 3.9 vs. 2.3 mg/L). There was a modest correlation between higher AUC_0–12h_ levels and the development of neutropenia (GM: 65.9 vs. 44.8 mg/L), but this was not observed for anemia, thrombocytopenia or transaminitis.

## DISCUSSION

Currently, there is no consensus on how to manage CMV disease in extremely preterm infants.^30^ In our center, the treatment of CMV disease in extreme preterm or VLBW infants is recommended for symptomatic disease. We followed the established practice of using 6 mg/kg GCV twice daily or 16 mg/kg valGCV twice daily as outlined in previous literature that closely aligns with our cohort.^22,23^

Data on the relationship between PK and pharmacodynamics for preterm and VLBW infants on either GCV or valGCV is lacking. The adult GCV target AUC_0–12h_ of 40–60 mg × h/L for symptomatic CMV treatment has also been proposed for children.^31^ However, children generally do not achieve that target when using the registered pediatric dosages of GCV and valGCV.^27^ Specifically, for treatment of cCMV, a target AUC_0–12h_ of 27 mg × h/L, with a range of 20–55 mg × h/L, has been suggested representing the 90th percentile in the studied population.^32^ These targets are based on population means and no data included infants less than 32 weeks gestation or VLBW.

Furthermore, adult C_min_ (0.5–1.0 mg/L) and C_max_ (7–9 mg/L) GCV therapeutic drug concentration ranges have been suggested in the literature.^33^ However, these ranges have not been shown to correlate with efficacy and toxicity in infants. In contrast to the widely used 5 mg/kg twice daily GCV treatment dose in adults, a higher dosage of 6 mg/kg twice daily GCV has been deemed preferable in term infants based on a small PK study.^32^ Additionally, a 6 mg/kg GCV and a 16 mg/kg valGCV twice daily dose yielded comparable drug concentrations.^22^ While some studies suggest dosing of GCV and valGCV based on estimated glomerular filtration rate (eGFR) and BSA for children, the currently recommended GCV and valGCV dose is based on mg/kg body weight in infants due to unreliability of eGFR in infants.^31,33^ Nevertheless, these dose recommendations have not been validated for preterm and VLBW infants.

GCV undergoes renal elimination and thus clearance is influenced by eGFR.^34^ Rapid maturation of renal function would complicate dosing particularly for preterm infants where the renal function is even lower at birth compared to term neonates.^35^ Furthermore, immaturity of the gastrointestinal tract in preterm infants may also affect the bioavailability of valGCV. These factors complicate GCV and valGCV dosing in preterm infants.

Our findings suggest that treatment of symptomatic CMV disease in preterm infants less than 32 weeks and weighing less than 1.8 kg led to high C_min_ and C_max_ levels, and higher than the recommended upper limit range of AUC_0–12h_. We observed that the GM C_min_ was much higher in preterm infants compared with term infants, whereas the GM C_max_ was only slightly higher. This suggests that reduced renal clearance is the primary factor contributing to the higher AUC_0–12h_ observed in our population. The rapid increase of renal clearance of GCV with increasing age in preterm infants complicates TDM monitoring and dosing regimens may require significant adjustments in the first weeks of treatment. It is important to note that our AUC_0–12h_ calculations were based on 2 sample timepoints—C_min_ and an approximate C_max_. Consequently, this estimation might underestimate the actual AUC_0–12h_. Similarly, using a single time-point measurement such as C_min_ in a clinical setting would not accurately estimate total GCV exposure in preterm infants.

It is essential that future studies on the PK of GCV or valGCV consider multiple time-point samplings to allow for accurate estimation of drug exposure. This would allow a model-based precision dosing strategy to optimize valGCV and GCV dosing in this population. Due to evolving renal clearance, an individual-based TDM might be necessary. However, it is crucial to minimize the volume of blood drawn in preterm infants due to their limited total blood volume. Nonetheless, we currently lack evidence of any correlation between GCV PK parameters and efficacy, which is a critical factor to eventually determine the desired therapeutic level of this drug.

Adverse effects with GCV and valGCV were prevalent among infants in our study especially in infants with lower gestation and younger age at the start of treatment. Based on this observation, we hypothesized that reduced drug clearance in these infants increases the likelihood of developing drug toxicity. However, any potential association between drug concentrations and toxicity remains unclear due to underpowered study and limited availability of PK data among infants who did not report adverse effects. Nonetheless, akin to their term infant counterparts, we observed that drug toxicity among preterm infants to be reversible.

## CONCLUSION

This study lays the groundwork for a deeper understanding of the complexities of GCV and valGCV PK and pharmacodynamics in extremely preterm or VLBW infants. The PK data and toxicity profile suggest that the 6 mg/kg GCV and 16 mg/kg valGCV twice daily dosing regime is too high for preterm and VLBW infants. Given the heterogeneity in PK, we anticipate that multiple-dose brackets with increasing gestational age and weight may be necessary. Future research should focus on developing and validating dosing regimens through PK modeling and simulation, followed by prospective PK studies to confirm these findings.
